# Cellular Orientation on Repeatedly Stretching Gelatin Hydrogels with Supramolecular Cross-Linkers

**DOI:** 10.3390/polym11122095

**Published:** 2019-12-14

**Authors:** Dae Hoon Lee, Yoshinori Arisaka, Asato Tonegawa, Tae Woong Kang, Atsushi Tamura, Nobuhiko Yui

**Affiliations:** Department of Organic Biomaterials, Institute of Biomaterials and Bioengineering, Tokyo Medical and Dental University (TMDU), 2-3-10 Kanda-Surugadai, Chiyoda, Tokyo 101-0062, Japan; lee.org@tmd.ac.jp (D.H.L.); tonegawa.org@tmd.ac.jp (A.T.); kang.org@tmd.ac.jp (T.W.K.); tamura.org@tmd.ac.jp (A.T.); yui.org@tmd.ac.jp (N.Y.)

**Keywords:** gelatin, polyrotaxane, hydrogels, oriented cells

## Abstract

The cytocompatibility of biological and synthetic materials is an important issue for biomaterials. Gelatin hydrogels are used as biomaterials because of their biodegradability. We have previously reported that the mechanical properties of gelatin hydrogels are improved by cross-linking with polyrotaxanes, a supramolecular compound composed of many cyclic molecules threaded with a linear polymer. In this study, the ability of gelatin hydrogels cross-linked by polyrotaxanes (polyrotaxane–gelatin hydrogels) for cell cultivation was investigated. Because the amount of polyrotaxanes used for gelatin fabrication is very small, the chemical composition was barely altered. The structure and wettability of these hydrogels are also the same as those of conventional hydrogels. Fibroblasts adhered on polyrotaxane–gelatin hydrogels and conventional hydrogels without any reduction or apoptosis of adherent cells. From these results, the polyrotaxane–gelatin hydrogels have the potential to improve the mechanical properties of gelatin without affecting cytocompatibility. Interestingly, when cells were cultured on polyrotaxane–gelatin hydrogels after repeated stress deformation, the cells were spontaneously oriented to the stretching direction. This cellular response was not observed on conventional hydrogels. These results suggest that the use of a polyrotaxane cross-linking agent can not only improve the strength of hydrogels but can also contribute to controlling reorientation of the gelatin.

## 1. Introduction

Gelatin is the physical and chemical denaturation product of collagen [1,2] and possesses several advantages as biomaterial [3,4,5]. For instance, gelatin has low antigenicity [6,7], high bioresorbability [8,9], and is relatively easy to chemically modify [10,11,12]. However, gelatin is difficult to use in specific in vivo affected areas where stress is repeatedly loaded, since it has a significant strength limitation. Various coupling or cross-linking agents such as glutaraldehyde (GA), formaldehyde, and methacryloyl derivatives have been used to strengthen the hydrolytic and enzymatic stabilities [13,14] and the mechanical properties of gelatins. Although it has been reported that GA treatment increases the mechanical strength and resistance to degradation of gelatin [15,16], the cytocompatibility of GA-treated gelatin tends to be reduced [17,18,19]. As a result, chemical treatments reduce the usefulness of gelatin as a scaffold for tissue regeneration and drug delivery applications.

**Figure 1:**
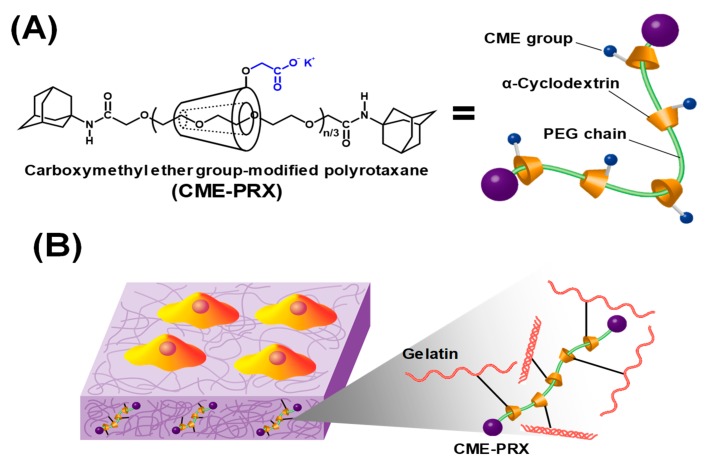
Schematic illustration of (**A**) CME-PRXs and (**B**) cell cultivation on gelatin hydrogels cross-linked by CME-PRXs. CME-PRX is supermolecule composed of α-CDs and PEG capped with bulky molecules. CME groups were modified to PRXs.

To solve this problem, we have focused on hydrogels based on polyrotaxanes (PRXs) [20]. PRX is a supermolecule formed by the self-assembly of linear polymers (e.g., polyethylene glycol (PEG)) and cyclic molecules (e.g., α-cyclodextrin (α-CD), and exhibits a mechanically interlocked structure in which many cyclic molecules are threaded on the polymers (Figure 1A) [21,22]. Ito and co-workers have reported that the use of PRX cross-linkers comprised many acrylate-modified α-CDs and PEG improve the mechanical properties of hydrogels [23]. When the hydrogel is deformed by applying external force, the threading α-CD sites, which act as cross-linking points for the hydrogels, can move along the PEG chains, thereby preventing stress concentration in hydrogels. We have also previously demonstrated the mechanical reinforcement of gelatin hydrogels using a PRX-based cross-linker in a hydrated state [24]. Conventional hydrogels cross-linked with *N*-hydroxysuccinimide (NHS) and 1-ethyl-3-(3-dimethylaminopropyl) carbodiimide hydrochloride (EDC) are easily broken because the cross-linking points are fixed, and loaded stress is focused by stretching [25]. By contrast, points in gelatin hydrogels cross-linked by PRXs can slide along the axle PEGs in the PRXs, resulting in elongation without the reduction of ultimate tensile strength values. Furthermore, we verified the unique properties of mechanical hysteresis loss for gelatin hydrogels cross-linked by PRXs. When we conducted a cyclic stretching–relaxation test, the gelatin hydrogels cross-linked by PRXs in a hydrated state showed lower hysteresis in stress–strain curves than gelatin hydrogels cross-linked with EDC/NHS. It is believed that the high hysteresis loss observed in gelatin hydrogels cross-linked with EDC/NHS is caused by higher energy dissipation, whereas low hysteresis loss in gelatin hydrogels cross-linked by PRXs might be caused by the sliding motion of threading α-CDs as cross-linking points [26]. Gelatin hydrogels with the mechanical property of low hysteresis loss have the potential to be implantable scaffolds that can adapt to the repeated-deformation of tissues such as blood vessels and muscles. However, their effects on cytocompatibility of the hydrogels such as cellular adhesion and proliferation have not been clarified. Furthermore, there is no literature on cellular responses on gelatin hydrogels with the mechanical hysteresis property before and after repeatedly stretching.

In the present study, the cytocompatibility of gelatin hydrogels cross-linked by carboxymethyl ether group-modified polyrotaxanes (CME-PRXs) was evaluated. Gelatin hydrogels were fabricated with or without the PRX cross-linkers with different numbers of threading α-CDs, and surface characterization, protein adsorption, and cell adhesion were tested. In addition, the cell attachment on gelatin hydrogels after cyclic stretching–relaxation treatment was investigated (Figure 1B).

## 2. Materials and Methods

### 2.1. Materials

Phosphate-buffered saline (PBS), Dulbecco’s-modified Eagle’s medium (DMEM), fetal bovine serum, penicillin, and streptomycin were purchased from Wako Pure Chemicals (Osaka, Japan). 2-(*N*-morpholino)ethanesulfonic acid (MES) buffer was purchased from Dojindo (Tokyo, Japan). EDC and NHS were purchased from Tokyo Chemical Industry (Tokyo, Japan). Gelatin powder was purchased from Nitta Gelatin (Osaka, Japan). Tissue culture polystyrene (TCPS) plate was purchased from Thermo Scientific (Waltham, MA, USA). Bovine serum albumin (BSA)-Alexa Fluor 488 conjugate was purchased from Molecular Probes (Eugene, OR, USA).

### 2.2. Preparation of Gelatin Hydrogels Cross-Linked by CME-PRXs

CME-PRXs were synthesized as described previously (Figure S1 from Supplementary Materials) [24,27]. In this experiment, two series of CME-PRXs with different α-CD threading ratios were used to fabricate gelatin hydrogels cross-linked by CME-PRXs, and the characterization of CME-PRXs was summarized in Table S1. CME-PRXs were labeled as CME-PRX-24% and CME-PRX-37%, corresponding to threading α-CD ratios of 24% and 37% in CME-PRXs, respectively, wherein the threading α-CD ratio is defined as the threading percentage of α-CDs in PRXs, assuming that one α-CD molecule forms an inclusion complex with two ethylene glycol units in the PEG axle. PEG chain (molecular weight: 35,000) can stoichiometrically thread 397 molecules of α-CDs at 100% threading ratio. However, it is difficult to collect 100% α-CD-threaded PRXs regardless of the feed ratio of α-CDs and PEG. The actual maximum ratio of threading α-CDs is approximately 40%. Further, PRX having a low ratio of threading α-CDs is difficult to purify and collect since it has the same polymer properties as unreacted PEG. Therefore, 24% and 37% of threading α-CDs in PRX indicate minimum and maximum ratio of threading α-CDs, respectively. The gelatin hydrogels cross-linked by CME-PRXs were prepared according to previous reports (Figure S2 from Supplementary Materials) [24,28]. In brief, gelatin powder (100 mg) was dissolved in 0.1 M MES buffer (1.0 mL) at 40 °C. CME-PRXs, EDC, and NHS were dissolved in 0.1 M MES buffer (1.0 mL) and activated to *o*-acylisourea or NHS-ester formation for 10 min at room temperature [29]. Activated CME-PRX solutions were then added to the gelatin solution (1:1 volume ratio) and stirred for 1 min at 40 °C. Finally, the mixture (2 mL) was set in a Teflon mold (width: 30 mm; length: 60 mm; height: 1 mm) and gelated to form amide groups between CME-PRX and gelatin at 4 °C, overnight. The fabricated gelatin hydrogels were washed in distilled water to remove the byproducts and unreacted EDC/NHS (1 h, four times). Because gelatin hydrogels have temperature dependency, pure gelatin hydrogels can be melted at 37 °C under the cell experimental condition. For this reason, we did not use pure gelatin as a control in this study.

### 2.3. Characterization of Gelatin Hydrogels Cross-Linked by CME-PRXs

A FT-IR spectrometer (Spectrum 100, Perkin Elmer, Waltham, MA, USA) was used to detect changes in the characteristic functional groups on the samples. All gelatin hydrogels were ground and blended with KBr to prepare pellets for measurements. All spectra were recorded in the frequency range of 500–4000 cm^−1^ at a resolution of 4 cm^−1^ and analyzed using Spectrum software (PerkinElmer). Static contact angle of air bubble was analyzed on each sample in water using a contact angle-measuring instrument (DropMaster DM-501) (Kyowa Interface Science, Saitama, Japan). A single air bubble (5 μL) was put on the surface and monitored using a charge-coupled device camera at 25 °C. The captured images were analyzed using FAMAS version 5.0.0 software (Kyowa Interface Science, Saitama, Japan). The morphology of dried gelatin hydrogels was visualized using Hitachi S-3400NX scanning electron microscopy (Hitachi, Japan). The samples were sputter-coated with 300 nm gold particles (SC-701AT, ELIONIX, Tokyo, Japan). All samples were examined using an accelerating voltage of 15 kV.

### 2.4. Preparation of Cell Culture Chambers

A cell culture chamber was fabricated in order to seed cells onto a hydrogel surface with a certain area and fix the stretched gelatin hydrogel. Tissue culture polystyrene (TCPS) plate was cut into a rectangular shape (width: 20 mm; length: 10 mm; height: 1 mm). Some of the rectangular-shaped TCPS chambers contained a round hole (diameter: 3.5 mm) in the center. Gelatin hydrogels (width: 7.5 mm; length: 30 mm; height: 0.5 mm) were sandwiched between original TCPS and holed TCPS plates; then, the two plates were fixed in place using plastic clips. The individual hydrogels were repeatedly stretched and then sandwiched between original TCPS and holed TCPS plates in order to fabricate cell culture chambers with stretched hydrogels. The process of repeatedly stretching is referred to as stretching–relaxation treatment. The cyclic stretching–relaxation was performed 20 times at a speed of 5 mm/s using a 2 N load cell. The stretching range of the hydrogels was 40 % strain.

### 2.5. Protein Adsorption Assay

Protein adsorption onto gelatin hydrogels cross-linked by CME-PRXs was evaluated according to previous reports [30]. Briefly, gelatin hydrogels cross-linked by CME-PRXs (width: 10 mm; length: 10 mm; height: 0.5 mm) were put onto 12-well plates. One hundred microliters of 0.5 mg/mL BSA–Alexa Fluor 488 conjugate was put on the surfaces of gelatin hydrogels cross-linked by CME-PRXs and incubated for 1 h at 37 °C. Gelatin hydrogels were then cross-linked by CME-PRXs and washed three times with PBS. Protein adsorption on the surface of gelatin hydrogels was recorded at 100 ms exposure time using a phase contrast microscope (IX71; Olympus, Tokyo, Japan) equipped with appropriate fluorescent filters (IX71; Olympus, Tokyo, Japan) and a dual CCD digital camera (DP80; Olympus, Tokyo, Japan).

### 2.6. Fibroblast Cultivation with Gelatin Hydrogels Cross-Linked by CME-PRXs

All hydrogels were sterilized by ultraviolet (UV) irradiation on a clean bench. In order to suppress the degradation of gelatin by UV light, the irradiation time was set to 30 min [31]. After sterilization, the hydrogels were washed three times with PBS. BALB/3T3 clone A31 mouse fibroblasts (RIKEN BioResource Center, Wako, Saitama, Japan) were seeded onto each hydrogel at a density of 0.5 × 10^4^ cells per cm^2^ and cultured using DMEM supplemented with 10% fetal bovine serum, 100 IU/mL penicillin, and 100 μg/mL streptomycin at 37 °C in a humidified atmosphere with 5 % CO_2_ for 2 days. The cell morphology was recorded using a phase contrast microscope (IX71; Olympus, Tokyo, Japan) equipped with a dual CCD digital camera (DP80; Olympus, Tokyo, Japan). The number of adhering BALB/3T3 cells on each hydrogel was counted in each image after 3, 6, 12, 24, and 48 h cultivations. In order to evaluate the orientation of the cells on the stretched hydrogels, the angle between the stretching direction of hydrogels and the spreading direction of cells was analyzed by ImageJ software (version 1.48, NIH, Bethesda, MD, USA). At least 187 cells from a minimum of eight images of each hydrogel were analyzed. 

### 2.7. Statistical Analysis

All data were analyzed using one-way analysis of variance in Excel software. A value of *p* < 0.05 was considered to indicate statistical significance.

## 3. Results and Discussion

### 3.1. Characterization of Gelatin Hydrogels Cross-Linked by CME-PRXs

**Figure 2:**
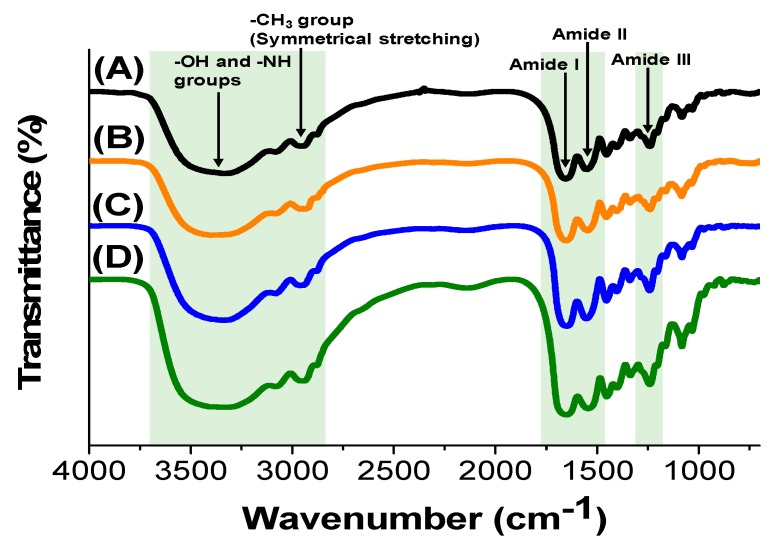
FT-IR spectra of (**A**) pure gelatin and gelatin hydrogels cross-linked by (**B**) EDC/NHS, (**C**) CME-PRX-24%, and (**D**) CME-PRX-37%.

FT-IR spectra of gelatin hydrogels cross-linked by CME-PRX-24% and CME-PRX-37% were analyzed to determine the chemical composition (Figure 2). As a control, gelatin hydrogels cross-linked with EDC/NHS were used. The peaks corresponding to O–H and N–H vibration were observed at 3700–3000 cm^−1^. The peaks of symmetrical stretching vibration of –CH_3_ groups were observed at 2940 cm^−1^. The peaks of amide I and II in gelatin were shown at 1700–1600 and 1590–1500 cm^−1^, respectively. The peak of amide III in gelatin was seen around 1200 cm^−1^ [32]. According to previous reports, we expected the symmetric vibration mode of carboxylate anions in CME-PRXs to be confirmed at 1413 cm^−1^ in FT-IR spectra [24,33]. It was difficult to observe a remarkable peak of carboxylate anions in CME-PRXs because the amounts of CME-PRXs in gelatin hydrogels were very small. However, we have previously clarified that gelatin is definitely cross-linked with CME-PRXs by the quantification of amino groups in gelatin hydrogels before and after the cross-linking with CME-PRXs [24]. These results indicate that the chemical composition of the surface in gelatin hydrogels were not remarkably changed by the type of cross-linking agents.

**Figure 3:**
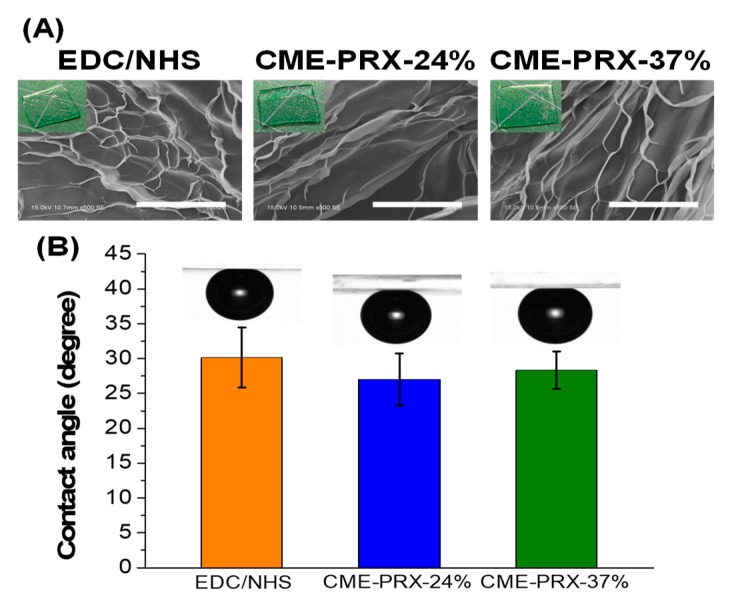
(**A**) Photographs of physical appearances and SEM images (scale bars: 100 μm) and (**B**) contact angles of air bubble on gelatin hydrogels cross-linked by EDC/NHS, CME-PRX-24%, and CME-PRX-37% (*n* = 3).

Figure 3 shows the physical appearances, morphologies, and contact angles of gelatin hydrogels cross-linked by CME-PRX-24%, CME-PRX-37%, and EDC/NHS. All hydrogels were transparent and had no noticeable turbidity (Figure 3A). Contact angle of the hydrogels was determined by an air bubble method, and the values were almost the same on each hydrogel (approximately 30 °) (Figure 3B). This result suggests that the chemical and physical properties of the surfaces in gelatin hydrogels cross-linked by CME-PRXs are almost equivalent to those cross-linked with EDC/NHS [7,34]. Based on SEM images of the gelatin hydrogels, the morphology of gelatin hydrogels was also similar regardless of the types of cross-linkers (Figure 3A). Accordingly, CME-PRX cross-linkers did not affect the chemical composition, wettability, or surface morphology of gelatin hydrogels compared to EDC/NHS.

### 3.2. Protein Adsorption Assay of Gelatin Hydrogels Cross-Linked by CME-PRXs

**Figure 4:**
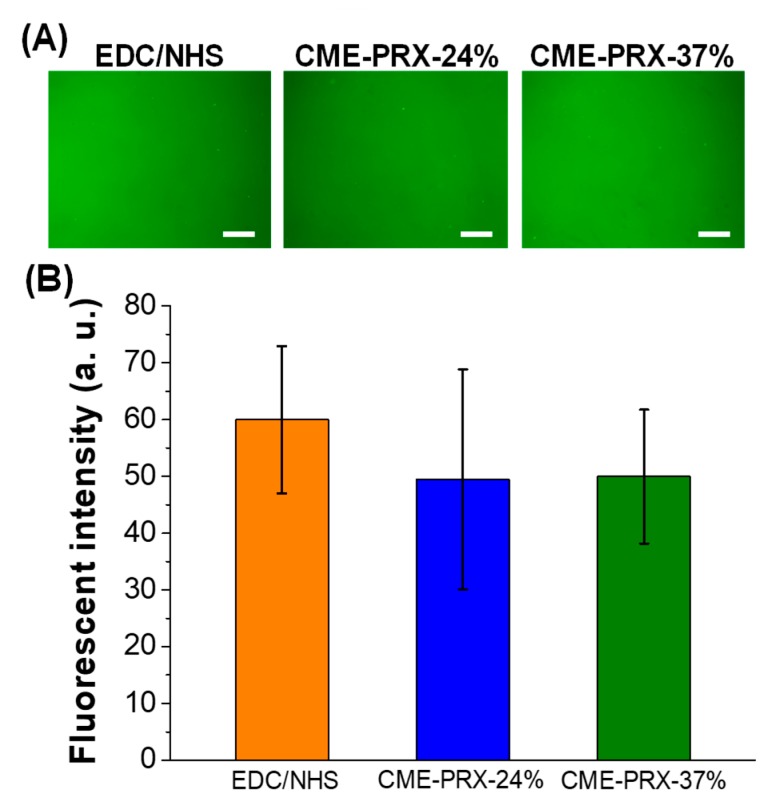
(**A**) Fluorescent images of gelatin hydrogels cross-linked by EDC/NHS, CME-PRX-24%, and CME-PRX-37% after the treatment of Alexa Fluor 488–BSA (0.5 mg/mL) for 1 h (scale bars: 500 μm). (**B**) Quantitative analysis of fluorescence intensity of adsorbed Alexa Fluor 488–BSA on the surfaces of gelatin hydrogels cross-linked by EDC/NHS, CME-PRX-24%, and CME-PRX-37% (*n* = 5).

Next, the adsorption of proteins on the surfaces of gelatin hydrogels cross-linked by CME-PRXs was investigated using fluorescent dye-conjugated BSA (Figure 4). BSA adsorbed on gelatin hydrogels cross-linked by EDC/NHS, CME-PRX-24%, and CME-PRX-37% was observed (Figure 4A). The fluorescence intensity of the surfaces of gelatin hydrogels after treating fluorescent BSA revealed that negligible difference in fluorescence intensity was observed (EDC/NHS: 60.0 ± 13.0; CME-PRX-37%: 50.0 ± 11.8; CME-PRX-24%: 49.5 ± 19.4) among all the gelatin hydrogels (Figure 4B). Because the chemical composition and wettability of the surface of gelatin hydrogels cross-linked by CME-PRXs were not changed, it was concluded that protein adsorption was not significantly changed on each hydrogel surface.

### 3.3. Cytocompatibility of Gelatin Hydrogels Cross-Linked by CME-PRXs

**Figure 5:**
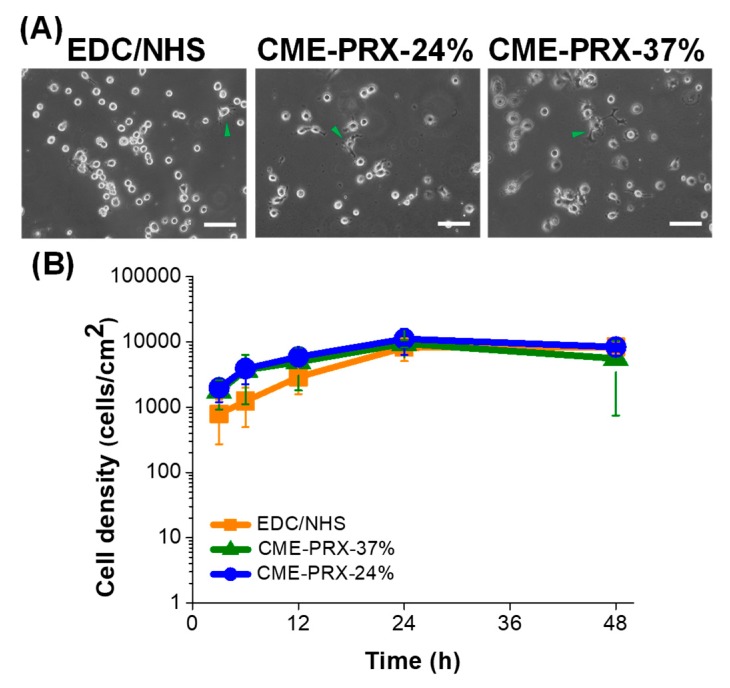
(**A**) Phase contrast images of BALB/3T3 cells after 6 h in culture on gelatin hydrogels cross-linked by EDC/NHS, CME-PRX-37%, and CME-PRX-24% (scale bars: 100 μm, arrows: spreading cell). (**B**) Proliferation of BALB/3T3 cells on gelatin hydrogels cross-linked by EDC/NHS, CME-PRX-37%, and CME-PRX-24% after 3, 6, 12, 24, and 48 h (*n* = 3).

To demonstrate the cytocompatibility of gelatin hydrogels cross-linked by CME-PRXs, the adhesion and proliferation of BALB/3T3 cells on gelatin hydrogels cross-linked by CME-PRXs was examined. After 6 h cultivation, the number of adherent cells on each gelatin hydrogel were counted from images (Figure 5A). The cell attachment was similar in all the gelatin hydrogels during cell cultivation, and most of the cells were attached within 24 h (Figure 5B). However, cell proliferation was not observed on all the gelatin hydrogels after 24 h. It has been previously shown that cell proliferation is inhibited when the strength of hydrogels is low [35]. On the other hand, there was no significant difference in the number of adherent cells between 24 and 48 h of cultivation on all the gelatin hydrogels. Because no reduction or apoptosis of adherent cells on the hydrogels was observed, the gelatin hydrogels cross-linked by CME-PRXs were suggested to have the same cytocompatibility as conventional gelatin hydrogels.

### 3.4. Orientation of Adhering Cells on Gelatin Hydrogels Cross-Linked by CME-PRXs

**Figure 6:**
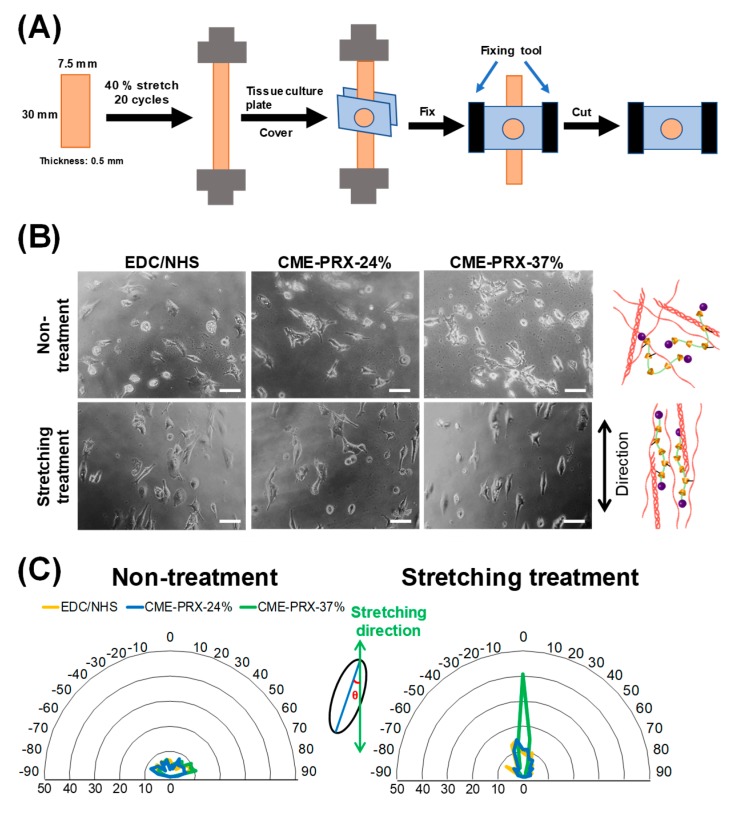
(**A**) Schematic illustration of culture-chamber fabrication for stretched gelatin hydrogels. (**B**) Phase contrast images of BALB/3T3 cells after 24 h in culture on gelatin hydrogels cross-linked by EDC/NHS, CME-PRX-37%, and CME-PRX-24% under normal and stretching states (scale bar: 100 μm). Schematic illustration of gelatin hydrogels cross-linked by CME-PRXs under non and stretching treatment. (**C**) Quantitative analysis of cellular orientation on gelatin hydrogels cross-linked by EDC/NHS, CME-PRX-37%, and CME-PRX-24% (*n* = 8; Round line: cell-oriented angle (degree); layer: percentage (%)).

In our previous study, the gelatin hydrogels cross-linked by CME-PRXs improve the mechanical properties, such as stretchability, toughness, and hysteresis. Therefore, it is expected that the cell attachment might be changed by applying repeated stretching–relaxation cycles or the orientation of the cells are altered by stretching. In order to investigate the influence of stretching of hydrogels on the cytocompatibility of gelatin hydrogels cross-linked by CME-PRXs, self-made culture chambers were fabricated as illustrated in Figure 6A to enable the evaluation of the cell attachment and orientation on the gelatin hydrogels in a stretching state. When BALB/3T3 cells were cultured on nonstretched and stretched gelatin hydrogels, the cell density on stretched gelatin hydrogels showed similar tendency to that on nonstretched gelatin hydrogels (Figure S3 from the Supplementary Materials). Cells on nonstretched gelatin hydrogels were spread randomly regardless of the types of cross-linkers. Interestingly, it was observed that the cells on stretched gelatin hydrogels cross-linked by EDC/NHS and CME-PRXs spontaneously spread along the stretching direction (Figure 6B). Since it was difficult to visually determine the cellular orientation from the photographs, image analysis was performed. When the percentage of stretching cells in the orientation angle range of −10° < θ < 10° was calculated, the percentage on the stretched gelatin hydrogels cross-linked by EDC/NHS, CME-PRX-24%, and CME-PRX-37% were 9%, 11%, and 41%, respectively (Figure 6C). These results suggest that repeatedly stretched gelatin hydrogels cross-linked by CME-PRX-37% remarkably direct the orientation of spreading cells along the stretching direction of the hydrogels. One possible explanation why cells are oriented is the reorientation of gelatin molecules in the hydrogels cross-linked by CME-PRX-37% [36,37]. In the case of conventional gelatin hydrogels cross-linked with EDC/NHS, the network of gelatin hydrogels is structurally destroyed by stress concentration after repeated stretching–relaxation. On the contrary, cross-linking by CME-PRXs provides the slidable cross-linking points in gelatin hydrogels. It is considered that the slidable cross-linking points contribute to promote the reorientation of gelatin molecules after stretching–relaxation for reducing the stress concentration. Furthermore, the rigidity of CME-PRX backbone is also involved in the reorientation of gelatin because it is reported that gelatin hydrogels containing rigid carbon nanotubes [38] and hydroxy apatites [2] show the molecular orientation by stretching. Generally, CME-PRXs with a high ratio of threading α-CDs have rigid backbone structure [39,40,41], which might better promote the reorientation of gelatin with respect to the stretching direction compared to CME-PRXs with a low ratio of threading α-CDs.

## 4. Conclusions

In the present study, we have shown that PRX cross-linkers have the potential to maintain the cytocompatibility of gelatin hydrogels. Interestingly, when cells were cultured on gelatin hydrogels cross-linked by CME-PRXs with a high ratio of threading α-CDs after stretching, the cells were spontaneously oriented along the stretching direction. This novel finding is not seen in other cross-linked gelatin hydrogels. It is suggested that PRX cross-linkers contribute to regulating the reorientation of the gelatin hydrogels. Furthermore, cyclic stretching–relaxation treatment can be expected to be applied as a technique for orienting soft materials like biological tissues. Since cellular orientation plays an important role in the function of myoblasts and vascular endothelial cells, gelatin hydrogels cross-linked by CME-PRXs could be used as a tissue regeneration scaffold that requires control of cellar orientation.

## Supplementary Materials

The following are available online at https://www.mdpi.com/2073-4360/11/12/2095/s1, Figure S1: Synthetic scheme of CME-PRXs; Figure S2: Schematic illustration of cross-linking mechanism between CME-PRX and gelatin; Figure S3: Cell density after 24 h cell cultivation on gelatin hydrogels cross-linked by CME-PRX-37% under stretching state; Table S1: Characterization of CME-PRXs.
