# Supplementary material for: Cellular Orientation on Repeatedly Stretching Gelatin Hydrogels with Supramolecular Cross-Linkers
Source: Polymers (Basel). 2019 Dec 14;11(12):2095. doi: 10.3390/polym11122095 (PMC6960878; doi:10.3390/polym11122095)
Supplement: Supplementary file 1 [file polymers-11-02095-s001.pdf]

polymers

# Supplementary materials

# Cellular Orientation on Repeatedly Stretching Gelatin Hydrogels with Supramolecular Cross-Linkers

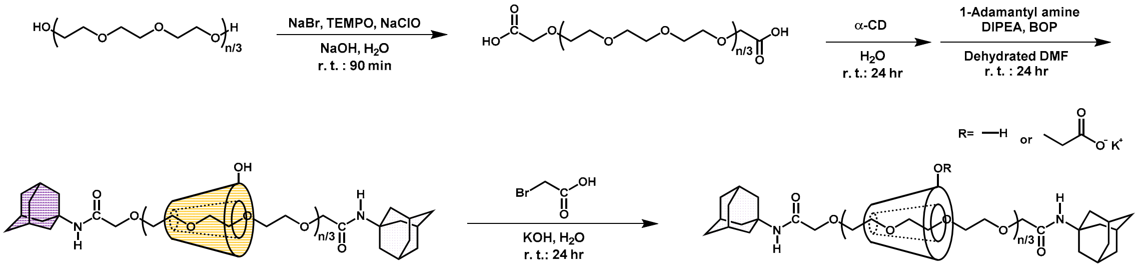


**Figure S1.** Synthetic scheme of CME-PRXs. (This figure was referred from [1].).

**Table S1.** Characterization of CME-PRXs. (This table was referred from [1].).

| Sample | $M_n$ of the PEG Axle | Number of Threaded α-CDs onto PRX[a] | Number of CME Groups on PRX[b] | $M_n$[c] |
|---|---|---|---|---|
| CME-PRX-24% | 35,000 | 96 (24.1 %) | 294 (3.06) | 157,000 |
| CME-PRX-37% | 35,000 | 147 (37.0 %) | 498 (3.39) | 227,000 |

[a]Determined by $^1H$ NMR in $D_2O$. The values in parentheses denote the threading percentage of α-CDs in PRX, assuming that one α-CD molecule forms an inclusion complex with two ethylene glycol units in the PEG axle. [b]Determined by $^1H$ NMR in $D_2O$. The values in parentheses denote the average number of CME groups per threaded α-CD in CME-PRXs. [c]Calculations based on the chemical composition of the CME-PRXs determined by $^1H$ NMR.

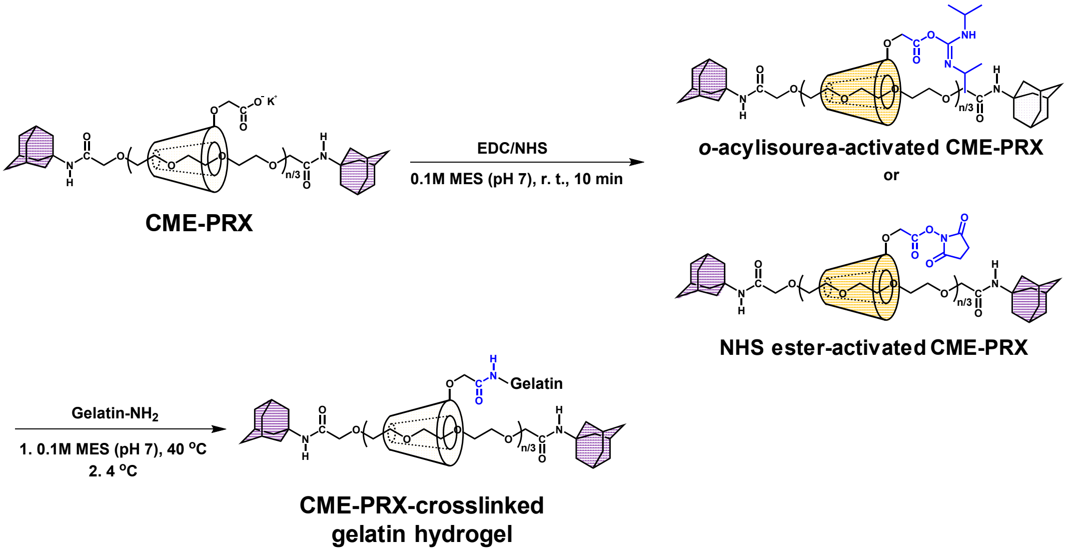


**Figure S2.** Schematic illustration of cross-linking mechanism between CME-PRX and gelatin.

## S1. Attached cell number on gelatin hydrogels cross-linked by CME-PRXs under stretching state.

To determine effect of cell attachment by stretched gelatin hydrogels cross-linked by CME-PRXs, attached cell number on gelatin hydrogels at 24 h under stretching state was investigated (Figure S3). The tendency of cell adhesion on gelatin hydrogels cross-linked by CME-PRX-37% under stretching state was similar to those of normal state. It was expected that the cell attachment by stretching mode of gelatin hydrogels was not affected because of high softness of gelatin hydrogels cross-linked by CME-PRX-37% in spite of stretching of hydrogels. From this result, it was demonstrated that stretched gelatin hydrogels cross-linked by CME-PRX-37% was not affected to cell attachment and cells on stretched gelatin hydrogels were aligned along with stretching direction.

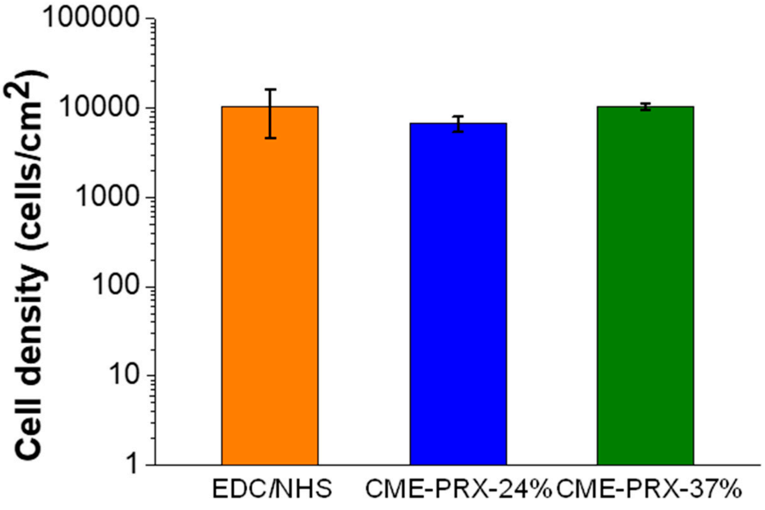


**Figure S3.** Cell density after 24 h cell cultivation on gelatin hydrogels cross-linked by CME-PRX-37% under stretching state (n = 3).

## S5. Reference

1. Lee, D.H.; Tamura, A.; Arisaka, Y.; Seo, J.-H.; Yui, N. Mechanically Reinforced Gelatin Hydrogels by Introducing Slidable Supramolecular Cross-Linkers. *Polymers* **2019**, *11(11)*, 1787.
